# An Uncommon Rash in the Emergency Department: Sporothrix schenckii

**DOI:** 10.7759/cureus.16125

**Published:** 2021-07-02

**Authors:** Dipal Shah, Alison E Kim, Samyr Elbadri, Bobby Desai, Latha Ganti

**Affiliations:** 1 Emergency Medicine, Ocala Regional Medical Center, Ocala, USA; 2 Emergency Medicine, University of Central Florida College of Medicine, Orlando, USA; 3 Public Health, Brown University, Providence, USA; 4 Emergency Medicine, Envision Physician Services, Plantation, USA; 5 Emergency Medicine, Hospital Corporation of America (HCA) Healthcare Graduate Medical Education Consortium Emergency Medicine Residency Program of Greater Orlando, Orlando, USA

**Keywords:** cutaneous manifestations, zoonosis and public health, sporothrix schenckii, emergency medical service, skin blister, non-healing ulcer

## Abstract

The authors present a case of *Sporothrix schenckii* diagnosed in the emergency department, based on a thorough history. The patient presented with skin nodules that had spread proximally up the arm in various stages of healing. He reported minimal pain for the unhealed ulcer and no pain for the healing ulcers, and no other concerning symptoms. The history of a thorn prick followed by the initial red nodule on the forearm has led to the diagnosis - as it was consistent with the classic presentation of lymphocutaneous sporotrichosis. A high index of suspicion and carefully noting occupational history is required for a diagnosis of sporotrichosis. Clinicians should recommend long sleeves and gloves to their patients when they are handling soil.

## Introduction

Sporotrichosis is an infection acquired by the fungus *Sporothrix schenckii* [1]. This causative agent thrives on a variety of thorny bushes and plants and is found in soil as well [2]. The hyphal form of the fungus occurs below temperatures of 37^º^C, but it will exist as a budding yeast above 37^º^C [3]. The morphology is relevant to identifying the transformation from mold to yeast [3]. There are several different strains of *S. schenckii*. *S. schenckii* complex is most commonly found in America, Asia, and Africa [3]. The typical presentation of the skin infection is that of a red nodule after trauma from rosebush thorns or wood splinters. The typical incubation period is up to three months. It begins as a papule, which then changes to a violaceous lesion. Following the solitary lesion, other lesions develop along the proximal lymphatics, which produce the classic lymphocutaneous form of the disease [3]. Although less common, mucosal, cutaneous disseminated, and extracutaneous forms have also been described [4]. These lesions can initially be misdiagnosed as bacterial in nature; the diagnosis becomes more evident when the patient complains of persistent ulcers on the hands and arms. Risk factors that contribute to the disseminated disease include immunocompromised individuals and patients with chronic obstructive pulmonary disease, alcohol use disorder, and diabetes mellitus [3]. Immunosuppressed patients are more susceptible because their cellular and molecular defects of the immune responses cause a predisposition to systemic fungal infections; this has been observed in co-infected HIV/*S. schenckii* patients [4].

## Case presentation

The patient is a 76-year-old male with a past medical history of hypertension and coronary artery disease, presenting with a non-healing red ulcer on his left forearm. He initially noticed a red nodule on his distal left arm one month prior and disregarded it, then describes multiple red nodules appearing and tracking proximally up his arm. He reports approximately 20 nodules that formed and began to moving up to his upper arm. On questioning, he does recall being pricked by a thorn on his Bougainvillea bush several days prior to the formation of the first nodule. Most of his lesions have begun to resolve on presentation to the ED; however, the first lesion is still present as a red ulcer that has not been improving. He describes a film forming over the non-healing ulcer that he has been wiping off with a dressing. He endorses minimal pain over the non-healing ulcer and no pain over the other healing ulcers. He also denies other symptoms such as fever, chills, cough, nausea, vomiting, diarrhea, shortness of breath, chest pain. He denies neurological deficits, numbness, or tingling.

Vital signs are within normal limits, with temperature 36.9ºC, heart rate 56, blood pressure 123/71 mmHg, respiratory rate 16, and oxygen saturation 98% on room air.

**Figure 1:**
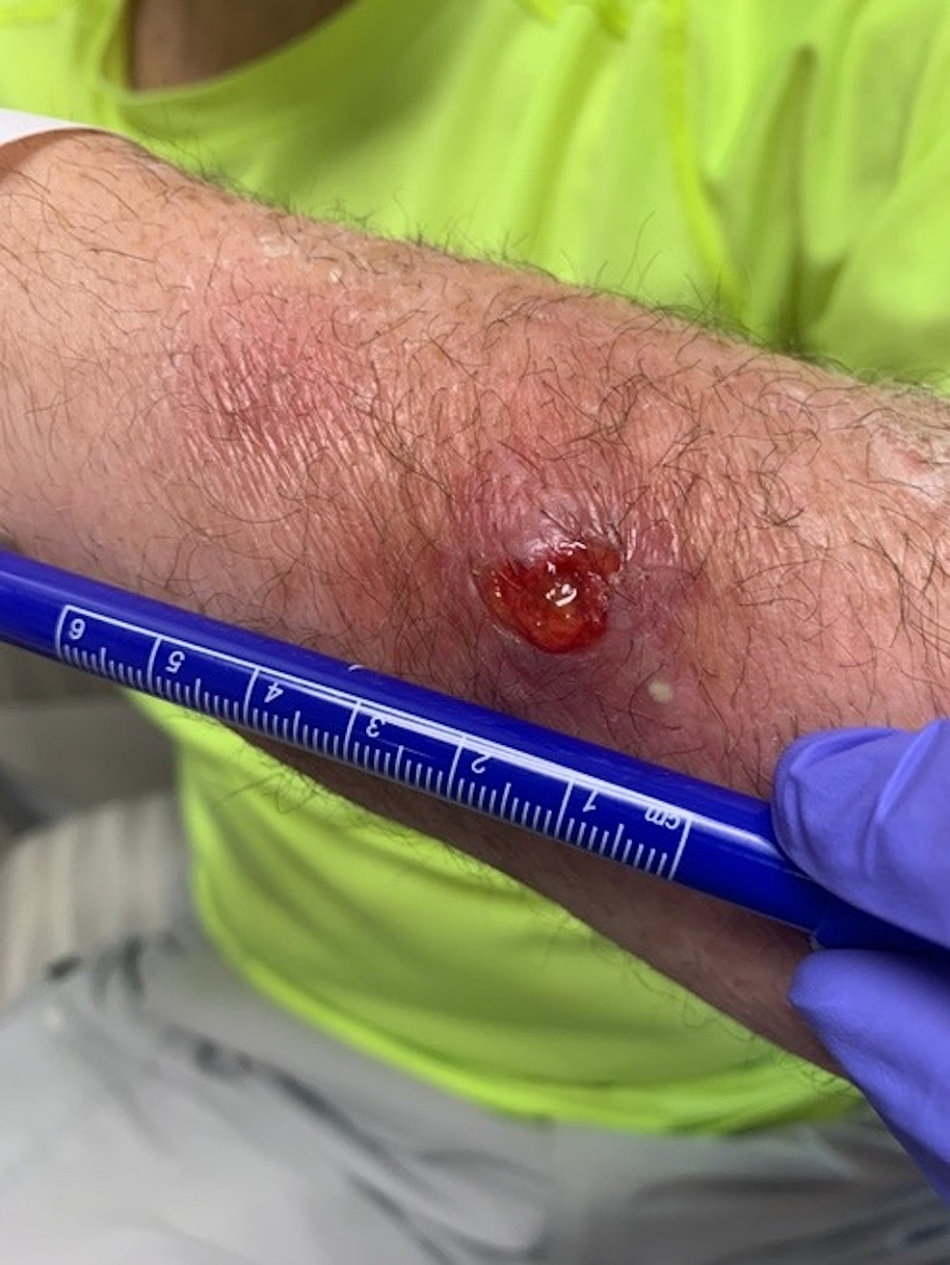
Clinical photograph depicting the length of one of the non-healing ulcers

A physical exam is notable for an erythematous raised nodule over the distal left forearm with no drainage, purulence, and mild tenderness on palpation. Crusted healing ulcers were noted proximally with no significant tenderness, erythema, and drainage (Figure 1). The physical exam is otherwise unremarkable.

The patient is educated on the lesions potentially taking months to resolve and is discharged on 200mg itraconazole daily with primary care physician follow-up, as treatment may be needed for over six months.

## Discussion

The patient is suspected of having lymphocutaneous sporotrichosis. Other forms of sporotrichosis include pulmonary, osteoarticular, meningeal, and disseminated sporotrichosis. The history of a thorn prick followed by the initial red nodule on the forearm is consistent with the classic presentation of lymphocutaneous sporotrichosis. The resulting lesions spreading proximally up the arm are also consistent with what is known as “sporotrichoid spread” or “nodular lymphangitis,” where similar lesions spread along lymphatic channels proximal to the original lesion. The differential includes skin abscesses or cellulitis and other systemic forms of sporotrichosis. These are less likely due to the classic presentation of lymphocutaneous sporotrichosis on history and physical exam and the absence of systemic signs such as fever, shortness of breath, and cough.

The diagnosis of sporotrichosis demands a high index of suspicion and taking a careful occupational history. Differential diagnoses can include necrotizing cellulitis, mycobacterial infections, such as *Mycobacterium ulcerans*; protozoal infections, such as leishmaniasis; or viral infections [5]. Inoculation most commonly occurs through cutaneous trauma from plants or as a zoonosis via infected animals such as cats or armadillos [1]. Sporotrichosis will initially appear as papules or pustules and subsequently morph into ulcerated nodules that involve local lymphatics [3]. If the abscess does not drain, it can wrongfully be a target for surgery [6]. One of the main risk factors is occupational exposure. Most reports indicate that two-thirds of sporotrichosis cases are young adults, mainly between the ages of 16 and 35 years old. Only one-third of cases occur in children, 5-15 years old [7]. Classified as a fungus, the gold standard for diagnosis is fungal culture and the treatment of choice is itraconazole [3].

Detecting the etiologic agent through histopathological means is often difficult in most sporotrichosis diagnoses because of a low fungal load and a copious number of cells that are seldom found in tissue. The fungus is usually present only in small numbers. Consequently, isolating the fungus in culture is sometimes difficult, but it may be necessary to differentiate between various species as they respond differently to antifungal therapy [8]. Although the first treatment of choice is itraconazole, for cutaneous-lymphatic and cutaneous-fixed sporotrichosis, potassium iodide is reported to have a favorable response when administered in diluted solutions or drops of saturated solution [7]. *S. schenckii* is temperature sensitive, and thus, thermotherapy or hyperthermia with temperatures of 45ºC, as well as hot baths at 45ºC for 15 to 20 minutes, two to three times a day, have also been reported as treatments with significant improvement. Evidently, temperatures above 42ºC often inhibit the fungus’ growth [7]. Therapy that relies on temperature should not be implemented alone, rather it is adjuvant therapy for localized cases or cutaneous-lymphatic sporotrichosis [7].

A recent increase of sporotrichosis among dense urban areas indicates a change in epidemiological patterns [4]. Despite the fungus’ transmission from the soil, vegetables, and wood indicating an association with rural work, physicians must be open to the diagnosis of sporotrichosis among patients in urban areas. Hypotheses that explain this trend include rises in temperature and humidity due to climate change, which favors fungal growth and the increase of dispersers through domestic animals contracting sporotrichosis. Correspondingly, cases linked to scratching and biting by domestic felines have increased, especially in Brazil. As a result of the disease no longer solely linked to adult male-dominated occupations such as farming, it is characterized as predominantly a zoonosis and affects a more diverse group of individuals. The gender ratio is 1:1 for all types of sporotrichosis, but there exists a slight male predominance especially for most cutaneous-disseminated cases. Exceptions include the zoonotic epidemic in Rio de Janeiro where the ratio was 2:1, female to male, due to a greater number of housewives [7]. In the United States, *S. brasiliensis* is the predominant etiological agent in cats.

## Conclusions

After a careful examination, it can be concluded that the non-healing ulcer, similar lesions throughout his arm, and recall of a thorn prick are consistent with lymphocutaneous sporotrichosis and that itraconazole is an effective treatment for this patient. This case demonstrates the importance of recognizing sporotrichosis in the Emergency Department because, without a careful history, the patient may be mistakenly diagnosed with a bacterial infection. If prescribed antibiotics, the course of the infection may be unnecessarily prolonged. However, due to variations in the geographical area of transmission, physicians should consider other skin and mucosal lesions such as cutaneous leishmaniasis, tuberculosis, leprosy, and some neoplastic and bacterial lesions. As demonstrated by the patient’s vulnerability to a thorn prick, clinicians should educate patients to wear long sleeves and gloves when handling soil and be cautious of transmission from domestic animals.
